# Correction: The Minimal Phenomenal Experience questionnaire (MPE-92M): Towards a phenomenological profile of “pure awareness” experiences in meditators

**DOI:** 10.1371/journal.pone.0314290

**Published:** 2024-11-18

**Authors:** Alex Gamma, Thomas Metzinger

The Data Availability statement for this paper is incorrect. The correct statement is: The data, documentation and all language versions of the MPE-92M questionnaire are publicly available as a ZIP-file on the Open Science Framework website. URL: https://osf.io/xerhg/download
