# Supplementary material for: The Minimal Phenomenal Experience questionnaire (MPE-92M): Towards a phenomenological profile of “pure awareness” experiences in meditators
Source: PLoS One. 2021 Jul 14;16(7):e0253694. doi: 10.1371/journal.pone.0253694 (PMC8279394; doi:10.1371/journal.pone.0253694)
Supplement: Attachment — Submitted filename: Response to reviewers.docx [file pone.0253694.s001.docx]

**PONE-D-20-34727**
“The Minimal Phenomenal Experience questionnaire (MPE-92M): Towards a phenomenological profile of “pure awareness”-experiences in meditators”

**Response to reviewers**

**Reviewer 1**

| **Reviewer comment** | **Response** |
| --- | --- |
| • 117: Minimalist idealization – terminology is not clear to general readership and non-philosophers, perhaps define | This is a very good point. We’ve changed the part starting at line 125 to read:  “(…) the idea is to develop a heuristic strategy of minimalist idealization, by de-scribing the target phenomenon in an uncluttered way, abstracting away from everything that is not an essential feature of the core explanandum [10]. For the target of phenomenal consciousness, does consciousness *per se* have a distinct experiential character? Does it ever occur in isolation, and can it be described in a conceptually precise manner? Part of the idea is to look for the decisive explanatory and computational factors by first attempting to construct exactly solvable minimal models ([10], p. 37). Such minimal models do not aim at maximal fidelity or completeness; they are not yet mappings to fine-grained functional mechanisms, and in this sense, they are idealizations.”  We hope this clarifies the terminology. |
| • 116 – 125: rationale for this second goal (minimal model explanation for conscious experience) is clearer in MPE paper (ref 10) – worth considering adding note on parsimony of such approach to clarify why this is a theoretical target | Thank you for the pointer. We wanted to keep this as short as possible but have now rewritten the whole paragraph to make all of this much clearer, thinking of a wider interdisciplinary audience. Drawing on the style and structure of previously published work it now reads:  “However, they can be crucial in generating a “minimal model explanation” [11,12]. Minimalist idealization proceeds by means of: constructing and further investigating a parsimonious model (…)  (…) Here, constructing such a minimal model would amount to eliminating superfluous details by extracting only the explanatorily relevant causal structure underlying the experience of awareness *per se*.” |
| • 180: Total usable responses were just 38.7% - the ‘Participants’ section (168) outlines minimum requirements, but doesn’t comment on why? Is there a limitation here around survey length and why there were so many incomplete responses? Consider adding to discussion of response drift (907) | Thank you for raising the issue. We have added a short discussion of the high number of incomplete responses to the discussion on representativeness: „The high number of incomplete responses (over 60%) also indicates a massive self-selection process, probably driven in part by the large number of questionnaire items, which may have been offputting.” |
| 210: typo – I think this should say table 6 | Thank you! We have corrected this. |
| • 248: does not mention whether control items (42 & 66) indicated reliable responses. This is discussed at 424 – although the authors do not suggest whether there was a criterion to exclude respondents based on responses. i.e. if there was a cut off for differences on control items over some arbitrary number. This is discussed in Limitations (914) and reasons for differences are mentioned with the salient point being that items were not identical. However, it is not acknowledged why no data was excluded on basis of the control items. Presumably, it would have impacted sample size. Consider adding something on this to fully acknowledge the limitation | Thanks for alerting us to this omission. We’ve added a passage to the limitations section discussing the issue of excluding subjects: “We did not exclude any participants based on the difference in their control item scores. We are generally very cautious about excluding participants and prefer to err on the side of inclusion unless there is a compelling rationale for doing otherwise. Also, a non-arbitrary exclusion criterion is hard to find in this case. But most importantly, a single pair of control items is much too noisy a criterion to offer a sound basis for data exclusion.” |
| • 619 -622: consider rewording for clarity: ‘… we can sometimes actively direct introspective attention to the quality of consciousness as such and we can distinguish possible states by the degree of actually ongoing introspective access to this quality.’ Bold part does not read well and likely to cause confusion | Unfortunately, we do not know what the “bold part” refers to since there is no formatting present in the reviewer’s comments as we received them.  Nevertheless, we have rewritten the passage and created two separate sentences; it should be much clearer now:  “(…) we can sometimes actively direct introspective attention to the quality of consciousness *per se*. If this turns out to be correct, then one can distinguish different states by the degree of actually ongoing introspective access to precisely this quality.” |
| • Observation: 585 (factor 1) and 815 (factor 11) point to the overall mix of phenomenological states reported from respondents in this study. There are a mix of elements that pertain to the more traditionally understood experience of Pure Consciousness directly and others that seem to be as an aside or weaved into the target experience surrounding more commonplace meditative experiences such as being aware of time, effort, duration and agency. These suggest a conceptual point which may be in line with the notion of ‘Glimpsing’ whereby states of pure consciousness for some may be sandwiched by more usual phenomenology, or, as you have suggested, refer to a multidimensional space of possible conscious states, a space which may contain different points, regions or trajectories. Ethically it seems relevant to acknowledge something here – practitioners can be perturbed by states they are taught about in some traditions which often feel illusive and an important part of soteriological endeavours. Acknowledging that in a large sample, experienced meditators have varied experiences including these states and other more banal or commonplace states might provide some instructive benefit for teachers and students | That is an excellent point. It is one result of our study which should be explicitly mentioned. We have now added two long sentences around line 638 (which previously included only “All of this is part of a normal meditation experience, and to be expected.”):  “Our data and a qualitative assessment of verbal reports (to be published in a separate monograph) show how, in a large sample, even experienced meditators and committed practitioners have varied experiences, including frequent attentional lapses and other banal or commonplace states. This point also relates to the existence of very short spontaneous occurrences of MPE in everyday life, and to the micro-meditative technique of “glimpsing” outside of formal practice, in both of which states of pure consciousness are sandwiched by longer periods of more ordinary phenomenology.” |

**Reviewer 2**

| **Reviewer comment** | **Response** |
| --- | --- |
| A general theoretical question concerns the very definition of the term “pure consciousness” as awareness of itself: “can a conscious system be exclusively aware of awareness itself ?” line 109. It’s not clear here what is an “awareness itself”. Is it a sort of phenomenon dissociated from the experiencing subject, or is it more like a kantian object (in itself?). What are the motivations behind this definition? This is a key question because the entire paper structure, as well as the survey depends on this definition. P 12 line 172 | The concept of “awareness“ is a phenomenological one, and not a metaphysical one. In a Kantian sense, it is treated as a *phenomenon* and not as a *noumenon*. In modern terminology, the question is whether awareness itself has a “phenomenal character” of its own.  To make this as clear as possible, we have now edited line 109 as follows:  “: Phenomenologically, can a conscious system be exclusively aware of awareness itself? Please note how the concept of “awareness“ we use is a phenomenological one, and not a metaphysical one (which might involve treating awareness as a Kantian “thing in itself,” or even some mysteriously self-conscious homunculus looking at awareness itself, etc.). This evidence-based phenomenological approach implies the possibility of non-egoic self-awareness, i.e., an experience of awareness itself that is not even subjective in the sense of being tied to a consciously experienced first-person perspective or a personal-level self-model anymore (see the discussion of Factor 3 and 8 below). If so, would there be a specific form of phenomenal character, a specific experiential quality that is instantiated during such episodes of pure awareness? Is there something like the simplest form of conscious experience, and if so, what exactly are the conceptual criteria for phenomenological “minimality”? Neuroscientifically, what are the minimally sufficient correlates for MPE to occur in neurotypical humans?” |
| Connected with the comment above: p 13 line 197: how many participants failed to understand the term “pure awareness” as the Authors define? Did you keep a record of these participants? | The reviewer might have overlooked that this is already reported in the results starting on line 377. This section also presents an analysis on the differences between those saying they do not understand the term “pure awareness” in the way we intended and those saying they understand it. |
| - P 13 line 207 – to which contemplative traditions these advanced practitioners belong to? As this influenced the questionnaire structure indirectly. | We have now added this information: “…and with the help of advanced practitioners who mostly belonged to the Vipassana and Zen traditions (similar to the final study sample, see Table 1).” |
| - P 20 line 340: meditation is a vast program with important differences in traditions. What is the “other” here refers to? | We did not ask respondents to specifically name any other meditation techniques they may have practiced, but our list was designed to cover the major examples worldwide. |
| - P 40 line 678 “pure awareness knows itself timelessly” – I am struggling understanding this phrase. Is pure awareness a sort of homunculus that knows itself intrinsically? Is it epistemic or experiential stance? | Thank you very much for pointing out this opportunity to clarify the point, very helpful. The interesting discovery is that there is now empirical evidence for a *non-egoic* form of self-awareness (no little man in the head). In ancient contemplative traditions this has been described as “self-knowing timeless awareness” or as “self-cognizing wakefulness” (for example in the Tibetan *rang rig ye shes*). Our stance is of course one of evidence-based phenomenology, but these ancient traditions often have different philosophical stances, e.g. epistemic (as you say) or metaphysical. We have added two explanatory sentences:  “The interesting discovery is exactly that there is now empirical evidence for a non-egoic, homunculus-free form of self-awareness. (…) Our approach is one of evidence-based phenomenology, but in ancient contemplative traditions this state has for many centuries been described epistemically, as “self-knowing timeless awareness” or as “self-cognizing wakefulness” (for example, in the Tibetan notion of *rang rig ye shes*).” |
| - P 41 – line 710 NREM acronym stands for … | We have added the expansion of the acronym (“non-rapid eye movement”). |
| - P 42 – I would move the paragraph 735 earlier in order to follow the structure: “traditionally X means Y, but here we mean Z” | Well, the final paragraph itself does just this:  Sentence 1 gives the traditional meaning, sentences 2 and 3 gives a new conceptual model, ending the section.  Paragraph 2 differentiates the major two different kinds of phenomenology and connects from the concepts of “radiance”, “brightness”, etc.  In the first paragraph, as in all other subsections, we have first said what the Factor refers to in our own study. To make all of this fully congruent, we have now added a sentence in the section on Factor 8:  In our study, it refers to an experiential quality of “pure knowing” without a sense of self and without any object. The relevant items describe an uncontracted, unbounded, and non-dual experience of wakefulness without content, which is self-aware, knowing only itself in a way that lacks any markers of egoic self-consciousness like agency, ownership, and autobiographical narrative, or spatiotemporal self-location [27, 30]. |
| One general worry I have is that the sample size is way to heterogenous and undercontrolled to back up a specific claim. Perhaps the next step should divide the sample in (i) different contemplative practices traditions ; (ii) years of practice; (iii) school of practice; (iii) gender ; (iv) socio-economical background ; (v) history of mental distress and potential disorders; | The issue of homo- vs heterogeneity of samples is an important one. Here we aimed at sampling a wide variety of respondents (a) in order to evaluate whether there could be a common core of the experience of “pure awareness” in meditators of very different backgrounds, and, more specifically (b) to allow effects of theory contamination to cancel out instead of dominating the results. We agree with the reviewer that investigating homogenous subgroups is worthwhile for more specific questions we might have going forward. (As this comment did not come with a suggestion for change, no changes to the manuscript have been made in this case.) |
| One last worry concerns the outlining of positive only aspects of meditative practices. Or, it is well documented that these practices that aim at attaining a certain “pure awareness” can lead to negative experiences and even distress (see Lindahl & Britton 2019). The journey toward the “pure awareness” does not see straightforward and the questionnaire should include the “dark” side of the journey as well. | This is an important point. Jared Lindahl and Willoughby Britton and their work are well known to one of us, and we will include this aspect in the next version of the questionnaire. In addition we have added a reference right at the point where positive affect is mentioned. Thanks again! |
